# Community‐level plant–soil feedbacks explain landscape distribution of native and non‐native plants

**DOI:** 10.1002/ece3.3649

**Published:** 2018-01-18

**Authors:** Andrew Kulmatiski

**Affiliations:** ^1^ Department of Wildland Resources and the Ecology Center Utah State University Logan UT USA

**Keywords:** Common‐garden, exotic, field experiment, plant distribution, plant invasion, semiarid, shrub‐steppe

## Abstract

Plant–soil feedbacks (PSFs) have gained attention for their potential role in explaining plant growth and invasion. While promising, most PSF research has measured plant monoculture growth on different soils in short‐term, greenhouse experiments. Here, five soil types were conditioned by growing one native species, three non‐native species, or a mixed plant community in different plots in a common‐garden experiment. After 4 years, plants were removed and one native and one non‐native plant community were planted into replicate plots of each soil type. After three additional years, the percentage cover of each of the three target species in each community was measured. These data were used to parameterize a plant community growth model. Model predictions were compared to native and non‐native abundance on the landscape. Native community cover was lowest on soil conditioned by the dominant non‐native, *Centaurea diffusa*, and non‐native community cover was lowest on soil cultivated by the dominant native, *Pseudoroegneria spicata*. Consistent with plant growth on the landscape, the plant growth model predicted that the positive PSFs observed in the common‐garden experiment would result in two distinct communities on the landscape: a native plant community on native soils and a non‐native plant community on non‐native soils. In contrast, when PSF effects were removed, the model predicted that non‐native plants would dominate all soils, which was not consistent with plant growth on the landscape. Results provide an example where PSF effects were large enough to change the rank‐order abundance of native and non‐native plant communities and to explain plant distributions on the landscape. The positive PSFs that contributed to this effect reflected the ability of the two dominant plant species to suppress each other's growth. Results suggest that plant dominance, at least in this system, reflects the ability of a species to suppress the growth of dominant competitors through soil‐mediated effects.

## 1 INTRODUCTION

Plant–soil feedbacks (PSFs) have rapidly gained attention as a potential mechanism explaining plant abundance, coexistence, succession, and invasion (Bailey & Schweitzer, 2016; van Der Putten et al., 2013; van der Heijden, Bardgett, & van Straalen, 2008). Plant–soil feedback experiments typically measure the growth of a target plant on soils cultivated by conspecific (“self”) and heterospecific (“other”) plants (Bever, 1994; Brinkman, Van der Putten, Bakker, & Verhoeven, 2010; Reinhart & Rinella, 2016). Positive PSF results when a plant grows better on “self” than “other” soils. Negative PSF results when a plant grows better on “other” than “self” soils. Mathematical models suggest that positive PSFs will result in persistent monocultures, whereas negative PSFs will result in coexistence through species replacements (Bever, 1994; Bever, Westover, & Antonovics, 1997; Vincenot, Cartenì, Bonanomi, Mazzoleni, & Giannino, 2017). These model predictions, however, assume that plants are competitively equivalent. Because plants are rarely competitively equivalent and experiments rarely monitor multiple generations of plants, PSF model predictions are rarely tested directly (van Der Putten et al., 2013). Instead, some of the best support for PSF model predictions comes from correlations between PSF and plant abundance on the landscape, but even these correlative tests remain rare (Bennett et al., 2017; Klironomos, 2002; Mangan et al., 2010; Teste et al., 2017).

Whether or not PSFs encourage plant invasion has long been a central question in PSF research (Callaway & Aschehoug, 2000; Callaway, Thelen, Rodriguez, & Holben, 2004). It has been suggested that PSFs are less negative for non‐native plants due to belowground enemies release (van Grunsven et al., 2007; Kulmatiski, Beard, Stevens, & Cobbold, 2008; Reinhart & Callaway, 2006). However, evidence for the role of PSFs in invasions remains mixed (Bunn, Ramsey, & Lekberg, 2015; Chiuffo, MacDougall, & Hierro, 2015; Crawford & Knight, 2017; Levine, Pachepsky, Kendall, Yelenik, & Lambers, 2006; Meisner et al., 2014; Müller, Kleunen, & Dawson, 2016; Schittko, Runge, Strupp, Wolff, & Wurst, 2016; Suding et al., 2013). A recent lack in support for the role of PSF in plant invasions may be due, at least in part, to a reliance on greenhouse‐based PSF experiments that may encourage the growth of plant disease (Bauer, Mack, & Bever, 2015; van Der Putten et al., 2013; Harrison & Bardgett, 2010; van der Putten, Bradford, Pernilla Brinkman, van de Voorde, & Veen, 2016; Schittko et al., 2016). Alternatively, some non‐natives may succeed due to the use of “novel weapons” or pathogen accumulation (Callaway et al., 2004; Eppinga, Rietkerk, Dekker, De Ruiter, & Van der Putten, 2006). These belowground mechanisms can increase invasive plant growth and also produce negative PSF. For example, a non‐native plant may benefit from large soil pathogen populations if those pathogens decrease the growth of “other” plants more than they decrease “self” plants (Eppinga et al., 2006). Finally, it is likely that some invasive plants benefit from PSFs while others succeed for other reasons, such as disturbance or release from aboveground pests.

While the number of PSF studies has rapidly increased over the past ten years, most PSF experiments remain limited to short‐term (i.e., ~6 month) measurements of plant monoculture growth under greenhouse conditions (Bennett & Cahill, 2016; Heinze, Sitte, Schindhelm, Wright, & Joshi, 2016; Kulmatiski et al., 2008; Schittko et al., 2016). There are many reasons that PSFs may differ between greenhouse and field conditions (Ehrenfeld, Ravit, & Elgersma, 2005; van der Putten et al., 2016; Schittko et al., 2016). By adding small volumes of soil inoculum to sterile soils under warm, wet conditions often with fertilizer addition, greenhouse experiments are likely to encourage the growth of fast‐growing or fast‐moving microbial species and their predators (Hawkes, Kivlin, Du, & Eviner, 2013; Kardol, De Deyn, Laliberté, Mariotte, & Hawkes, 2013; Poorter et al., 2016). Similarly, most PSF experiments measure growth responses of plant monocultures (but see Casper & Castelli, 2007; Smith & Reynolds, 2012; Shannon, Flory, & Reynolds, 2012). It is not clear how mixed plant communities respond to different soil conditions although it has been suggested that competition in communities may exaggerate PSF effects (Hol, de Boer, ten Hooven, & van der Putten, 2013; Kardol, Cornips, van Kempen, Bakx‐Schotman, & van der Putten, 2007) or community interactions may result in species‐specific PSF responses that are different from monoculture PSF responses (Casper & Castelli, 2007; Hendriks, Mommer, de Caluwe, Smit‐Tiekstra, & van Der Putten, 2013). The need for research that measures PSFs in plant communities and over longer time periods is well recognized (Casper & Castelli, 2007; van Der Putten et al., 2013; Teste et al., 2017; Smith‐Ramesh and Reynolds, 2017).

The overarching objectives of this study were to (i) measure community‐level PSF for a native and a non‐native community using a seven‐year common‐garden experiment and (ii) test whether or not measured PSFs can help explain native and non‐native plant abundance on the landscape. I predicted that native and non‐native communities would realize positive PSF and that these PSFs would improve predictions of plant growth on the landscape. This is because native and non‐native plants on the landscape have been reported to create distinct and persistent communities, and positive PSF provides a mechanism for this pattern (Kulmatiski, Beard, & Stark, 2006). To test this prediction, the growth of a three‐species native plant community and a three‐species non‐native plant community were measured on both native‐ and non‐native‐cultivated soils. These soil treatments were created in a common‐garden over 4 years. Plant community responses were measured after three additional years of growth. Species‐level plant growth data were used to parameterize a PSF model of plant community growth (Kulmatiski, Beard, Grenzer, Forero, & Heavilin, 2016). Model predictions were compared to plant growth on the landscape determined from a vegetation survey.

## 2 MATERIALS AND METHODS

Research was conducted near Winthrop, Washington (48.481 N, −120.117 W; elevation 780 m), in the Methow valley on the Newbon soil series (coarse‐loamy, mixed mesic Typic Haploxerolls; Lenfesty, 1980). The biotic and abiotic conditions of the valley have been described elsewhere (Kulmatiski, 2006; Kulmatiski et al., 2006; Kyle, 2005). Briefly, annual precipitation (380 mm) falls mostly in the winter as snow and plant growth occurs primarily between April and July with limited growth in the Fall. Relative to long‐term mean annual precipitation, annual precipitation during this study was 25% smaller from 2007 to 2009 (281, 291, and 278 mm, respectively), 31% larger from 2010 to 2012 (522, 474, and 502 mm, respectively), and 32% smaller in 2013 (259 mm).

There are two common plant community types within the shrub‐steppe ecosystem that exists in the Methow valley: Fields that have never been tilled represent most of the land in the hilly landscape and are dominated by native plants. Most valley bottoms and benches are or have been used for agriculture and are dominated by non‐native plants (Kulmatiski, 2006). This research focused on three of the most common native species and three of the most common non‐native species in the never‐tilled and abandoned‐agricultural fields, respectively. The three natives were relatively long‐lived bunchgrasses. These three species, *Pseudoroegneria spicata, Festuca idahoensis,* and *Koeleria cristata* cover 18.9%, 2.9%, and 0.2% of the ground in never‐tilled fields, and together account for 41% of total herbaceous cover in these fields (Kulmatiski, 2006). The three non‐natives were a short‐lived grass (*Bromus tectorum*) and two short‐lived (typically 1–2 years), tap‐rooted forbs (*Centaurea diffusa,* and *Sisymbrium loeselii*). These species cover 4.5%, 5.1%, and 3.0% of the ground in abandoned‐agricultural fields, and together account for 23% of herbaceous cover in these fields (Kulmatiski, 2006). Some common plants were excluded from the experiment. The large native shrubs, *P. tridentata* and *A. tridentata* and the rhizomatous *Cardaria draba* were not used because their growth could not be constrained within 1.5 m^2^ experimental plots. *Poa bulbosa* is a dominant non‐native, but it would not establish in this experiment.

Soil traits on the landscape tend to differ more as a function of plant type than agricultural history. For example, soil organic matter in never‐tilled fields was found to be 53 g/kg under non‐native plants and 64 g/kg under native plants but soil organic matter did not differ between tilled and never‐tilled soils (Kulmatiski et al., 2006). Similarly, extractable inorganic N pools tend to be smaller under non‐natives (21 mg/kg) than under natives (28 mg/kg), and net N mineralization rates tend to be faster under non‐natives (267 mg m^−2^ day^−1^) than natives (210 mg m^−2^ day^−1^), but these traits do not differ as a function of agricultural history (Kulmatiski et al., 2006). Soils in surrounding fields are comprised of roughly 72% sand and 11% clay (Kulmatiski et al., 2006).

### 2.1 Plant–soil feedback experiment

**Figure 1:**
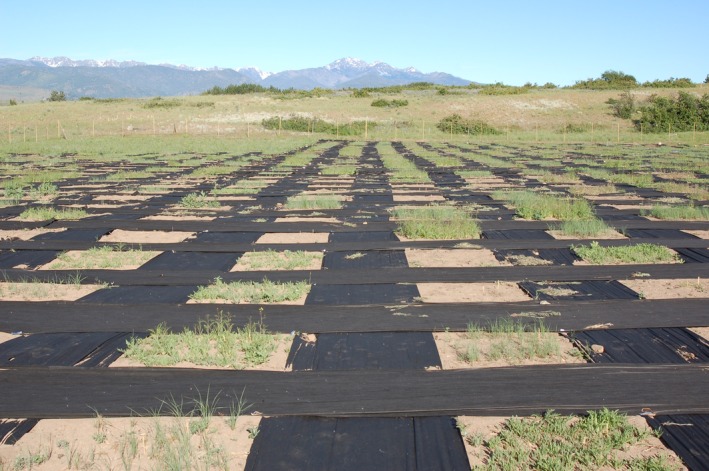
Photograph of the experimental plots during phase I of a seven‐year common‐garden plant–soil feedback experiment, Winthrop, WA, USA

**Figure 2:**
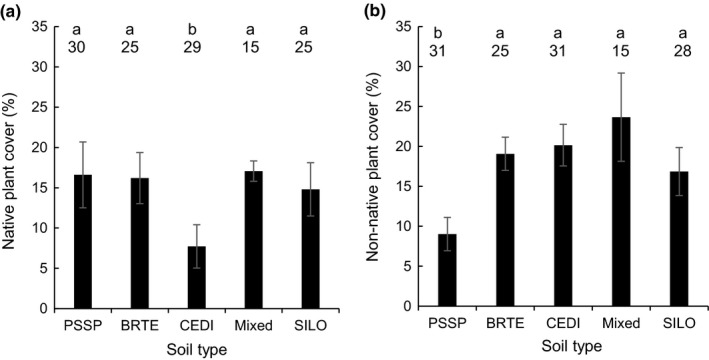
Percentage cover of (a) native and (b) non‐native plant communities on five different soil treatments. Soil treatments were created in a common‐garden experiment by growing target plant species for 4 years then removing vegetation. Native and non‐native communities were then grown for 3 years prior to measurement. Mean cover ± 1 *SE* shown. Bars with different lower case letters different at the α = .05 level. Numbers above bars indicate sample size. PSSP, *Pseudoroegneria spicata*; BRTE, *Bromus tectorum*; CEDI, *Centaurea diffusa*; Mixed, a naturally recruiting mix of species; SILO, *Sissymbrium loeselii*

Briefly, 372 plots (1.2 by 1.2 m) were planted with one of six plant species to create six target soil treatments (Figure 1). This sample size was designed to produce 32 replicate plots on each of six soil treatments for one, three‐species native community and one, three‐species non‐native community. However, because target plant growth did not attain a predetermined level of 65% of standing vegetation by the end of the soil‐cultivation phase, there were not 32 replicates of each plant community growing on each soil treatment. Notable, two of the native grasses failed to dominate plots. These plots were used to create soils cultivated by a mixture of native and naturally recruiting non‐native plants. As a result, the experiment included five soil types: one native soil, three non‐native soils, and one “mixed” soil. Actual sample sizes ranged from 15 to 31 on each soil type and are shown in Figure 2.

An abandoned‐agricultural field previously used to grow alfalfa (*Medicago sativa*) was used to establish a two‐phase, “self” versus “other” PSF experiment (Bever, 1994). Prior to Phase I, in October 2006, the weed seed bank in the top 10 cm of soil was removed by bulldozer. A 25 cm thick A2 layer remained below this removed layer (Lenfesty, 1980). Soils from a nearby native‐dominated field were mixed with equal amounts of sand from a nearby landslide to add roughly 6 cm of native soil inoculant. Sand was added to ensure better mixing of the native and non‐native inoculant. Native soil was collected from a field with 31% *Purshia tridentata,* 22% *P. spicata,* 19% *Balsamorhizae sagittata,* 4% *Artemisia tridentata,* 4% *Lupinus sericeus,* 2% *Lithospermum arvensis,* and 2% *B. tectorum* (Kulmatiski, personal observation). Several passes with a disk harrow to 15 cm was used to mix the added native and sand soils with soils from the experimental field. A grid of 1.2 m‐wide geotextile cloth was used to create 372, 1.2 m × 1.2 m plots. Each Fall from 2006 to 2009, 12 g of seed from each target species was planted in 62 replicate plots. Each summer, nontarget plants were removed by hand to maintain monocultures of target plants. In May 2010, all plots were surveyed. Plots where the target species did not represent 65% or more of standing vegetation were removed from the experiment. All *K. cristata* and *F. idahoensis* plots were removed because these species did not represent 65% of total cover. For the remaining species, 50–61 replicate plots were used in the experiment. Thirty of the *K. cristata* and *F. idahoensis* plots that demonstrated between 30% and 50% target plant growth were retained and included as “mixed” community plots. These plots contained a mix of target native plants and a variable mix of naturally recruiting non‐native plants. Beginning June 2010, all remaining quadrats were treated with a broad‐spectrum herbicide application (30 ml of Roundup^®^ herbicide, 0.2 kg active ingredient/ha). Two weeks later, standing vegetation was clipped by hand and left in the plot. Plots were revisited over the next several months and additional herbicide spot‐treatments and hand‐pulling were used in quadrats where regrowth was observed.

Phase II began October 2010. The three‐species native community and the three‐species non‐native community were planted on each of the five soil treatments cultivated in Phase I. Twelve grams of seed (4 g from each of three target species) was added to each plot. Nontarget species were removed by hand weeding during the 2011, 2012, and 2013 growing seasons. In June 2013, percentage cover of each target plant was estimated in each plot by two observers using visual estimation.

### 2.2 Landscape vegetation survey

To assess the landscape abundance of the target species, the 25 sites described in Kulmatiski (2006) were surveyed each June from 2007 to 2013. Each site contained four transects (50–100 m long) in an abandoned‐agricultural field and an adjacent never‐tilled field. In abandoned‐agricultural fields, two transects were located parallel to and either 5 or 50 m from historical tillage boundaries (−5 or −50 m). Similarly, in never‐tilled fields, two transects were located parallel to and either 5 or 50 m from historical tillage boundaries (5 or 50 m). Fifteen, 1 m^2^ quadrats were evenly spaced across each transect. The percentage cover by species was assessed visually in each quadrat. Visual estimates were well correlated (*R* ^2^ = .95) with 81‐point‐intersect estimates (Kulmatiski, 2006). The sites occurred over a 25 km stretch of the Methow valley and represented a 62‐year chronosequence of agricultural abandonment and so provided inference into long‐term patterns of native and non‐native abundance in the valley (Kulmatiski, 2006).

### 2.3 Model parameterization

The PSF model that best predicted plant community growth in Kulmatiski et al. (2016) was used (i.e., the “Pot‐Level‐K” model). Briefly, this logistic growth model is founded on three assumptions: Each plant creates a soil type, the growth of each soil type is a function of the abundance of the plant that creates that soil type and each plant grows at a rate that is specific to each soil type. Growth rates are derived from observed plant cover in the PSF experiment. Each plant is assumed to grow from seed (assumed to cover 0.004 m^2^ m^−2^) and time‐step‐specific growth rates were calculated for 55 time steps (i.e., roughly two‐day time steps for a 110 day growing season) as

$$\left({}^{55}\sqrt{\frac{F}{I}}\right)-1,$$

where *F *= final ground cover and *I *= initial ground cover. Plant growth in each time step was assumed an additive function of the proportion abundance of each soil type. The mean plus two standard deviations of total native or non‐native plant growth observed in the PSF experiment was used to estimate the carrying capacity for all native or all non‐native plants. These values were very similar for natives and non‐natives (i.e., 42% and 41% ground cover, respectively) and also similar to the ground cover observed in native and non‐native communities on the landscape (i.e., 43% and 38%, respectively; Kulmatiski, 2006).

### 2.4 Statistical and modeling analyses

Differences in total target native or non‐native plant cover among soil treatments in the PSF experiment were tested using a one‐way generalized linear model in a completely randomized design with “soil treatment” as the fixed effect (Proc Glimmix in SAS v 9.4). For the vegetation survey, differences in total target native or non‐native plant cover between abandoned‐agricultural and never‐tilled fields, and between distance transects were tested using a generalized linear mixed model in a two‐way factorial design. Fixed effects were plant origin (native or non‐native) and distance from tillage boundary (−50, −5, 5 or 50 m). Fields were random effects. Data from the 15 quadrats per transect and from the 7 years of the survey were averaged prior to analyses. Percentage cover values were arcsine square‐root transformed to better meet assumptions of homogeneity of variance and normality. Analyses performed using Proc Glimmix in SAS v 9.4 for Windows (SAS Institute, NC, USA).

### 2.5 Model execution

The goal of the model simulation was to isolate PSF effects from other effects that may determine plant abundance. To do this, soil treatments were assigned according to the landscape abundance of native and non‐native plants, and “propagules” were assigned equally for all species. To be clear, the model was initiated with soil treatments that reflected the landscape abundance of native and non‐native plants, but after the initial time step of the model simulation, the proportion of each soil type was determined by the relative abundance of each plant that grew in the previous time step. This can be considered to simulate a scenario in which all living vegetation was removed from the landscape and both native and non‐native propagules were added equally everywhere. More specifically, plant abundance data from the vegetation survey were used to estimate the relative abundance of native and non‐native soils. In abandoned‐agricultural fields, 50 m from tillage boundaries (−50 m) native plants represent 25% of plant cover and non‐native plants represent 75% of plant cover (Kulmatiski, 2006), so these soils were assumed to contain 25% native soils and 75% non‐native soils. Similarly, in abandoned‐agricultural fields, 5 m from tillage boundaries (−5 m), native plants represent 36% of plant cover so soils were assumed to be comprised of 36% native soil. In undisturbed fields, native plants represent 86% and 94% of plant cover 5 and 50 m from tillage boundaries (Kulmatiski, 2006). However, because plant growth rates on *F. idahoensis* and *K. cristata* soils were not available, all native soils were assumed to be cultivated by the dominant native, *P. spicata*. This was not likely to have large effects on results because *P. spicata* is a dominant plant, so most native soils were likely to become *P. spicata‐*cultivated soils during the model simulation.

The model was also executed without PSF effects (i.e., as a null model; Kulmatiski, Heavilin & Beard 2011). In the null model, each plant species had only one growth rate which was derived from the cover each plant attained on “self” soils (Kulmatiski et al., 2016; Kulmatiski, Heavilin & Beard 2011). Use of the null model allowed a comparison of model predictions with and without PSF effects. For both the PSF and null model, the model was executed for 165 days to simulate growth during the 3 years of Phase II in the field experiment.

## 3 RESULTS

In the PSF experiment, native plant cover differed among soil treatments (*F*_5,113_ = 6.32, *p *<* *.0001; Figure 2). This reflected the fact that native cover was 52% smaller on soils cultivated by *C. diffusa* than the rest of the soils (on average). Non‐native plant cover also differed among soil treatments (*F*_5,125_ = 3.65, *p *<* *.0076; Figure 2). This reflected the fact that non‐native cover was 55% smaller on soil cultivated by *P. spicata* than the rest of the soils (on average).

**Figure 3:**
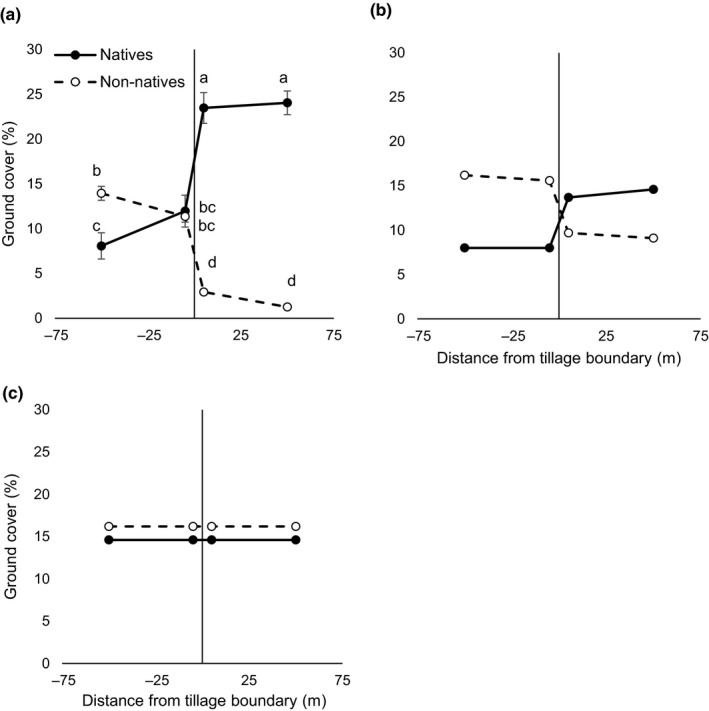
(a) Observed and (b and c) predicted abundance of three dominant native and three dominant non‐native plants (% ground cover) across historical tillage boundaries. Observed data represent the mean cover of the target species in 25 paired, randomly selected fields near Winthrop, WA, USA. (b) Consistent with observed plant growth, a plant growth model that included plant–soil feedback effects predicted that native plants would dominate on native soils and non‐native plants would dominate on non‐native soils. This model assumed that propagule pressure was equal for all species on all soils. (c) When PSF effects were removed from the model, non‐native plants were predicted to dominate the landscape. See Section 2 for model description. Negative *x*‐axis values indicate samples taken within abandoned‐agricultural fields and positive values indicate samples taken in adjacent undisturbed soils (Kulmatiski, unpublished). Values of plant cover represent the mean for 25 fields (±1 *SE*). Native and non‐native values within a distance category with an asterisk are different at the α = .05 level

For vegetation on the landscape, an interaction between plant origin and distance from tillage boundary (*F*_3,102_ = 23.94, *p *= <.001) reflected a switch in native and non‐native plant dominance between never‐tilled and abandoned‐agricultural fields. Target native cover was greater than target non‐native cover in never‐tilled fields, but target non‐native cover was greater than target native cover in the 50 m transects in abandoned‐agricultural fields (Figure 3a).

When plant growth in the PSF experiment was used to parameterize the PSF model, native plants were predicted to be more abundant than non‐native plants on native soils, and non‐native plants were predicted to be more abundant than native plants on non‐native soils (Figure 3b). When PSF effects were removed from this model, non‐native plants were predicted to be more abundant than native plants across the landscape (Figure 3c).

## 4 DISCUSSION

**Figure 4:**
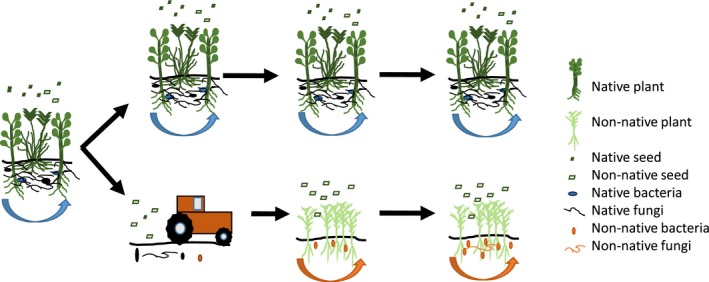
Conceptual diagram of a proposed multistep invasion process in the study system. (i) Soil disturbance caused by agriculture disrupts soil conditions that encourage native plant growth and discourage non‐native plant growth, (ii) agricultural abandonment allows the establishment of early‐successional, non‐native plants and (iii) once established, these species cultivate soil conditions that prevent native plant re‐establishment

Results provided clear evidence that PSF can help explain the distribution of native and non‐native plants on the landscape. Using a long‐term, common‐garden experiment, a native plant community was found to grow poorly on soils cultivated by the dominant non‐native plant, and a non‐native plant community was found to grow poorly on soils cultivated by the dominant native plant. In other words, both the native and non‐native plant communities realized positive PSFs. When these data were used to parameterize a plant growth model, native plants were predicted to dominate their own soils and non‐native plants were predicted to dominate their own soils. This prediction was consistent with patterns of plant abundance on the landscape: native plants dominate and are persistent on never‐tilled fields and non‐native plants dominate and are persistent on abandoned‐agricultural fields (Kulmatiski, 2006). Without PSF effects, the null model predicted that non‐native plants would dominate all soils, which was not consistent with plant growth on the landscape. Results suggest a multistep conceptual model of plant invasion in this system: (i) agriculture removes soil legacies that inhibit non‐native plant growth (i.e., *P. spicata* legacies), (ii) agricultural abandonment allows the establishment of early‐successional, non‐native plants and (iii) once established, these species, namely *C. diffusa,* create a soil that prevents native plant re‐establishment (Figure 4).

A previous study in nearby fields also reported positive PSFs for native and non‐native plants (Kulmatiski, 2006). That study, however, used a natural‐experiment approach that could not distinguish PSF from agricultural legacy effects. Here, a common‐garden experiment ensured that plant growth responses reflected PSF effects and not agricultural legacies.

This experiment was designed to produce a quantitative test of the role of PSF on the growth of two plant communities, but results were also consistent with common hypotheses regarding the role of PSFs in succession, invasion, and abundance (Callaway et al., 2004; Kardol et al., 2007; Klironomos, 2002). PSFs are generally believed to be more positive for late‐relative to early‐successional species (Bauer et al., 2015) and consistent with this, the native community demonstrated a positive PSF. Further, a paired experiment in the same field found a positive correlation between plant lifespan and PSF for native plants (Kulmatiski et al., in press). However, the non‐native community, which was comprised of short‐lived plants, also realized a positive PSF. This was not consistent with the idea that early‐successional species realize negative PSF, but was consistent with the idea that non‐native, particularly invasive species, benefit from positive PSF (Callaway et al., 2004; Kulmatiski et al., 2008; Maron, Klironomos, Waller, & Callaway, 2014; Reinhart & Callaway, 2006). This idea has been popular for more than 10 years (Levine et al., 2006; Reinhart, Packer, Van der Putten, & Clay, 2003), but several recent studies have failed to demonstrate positive PSFs for invasive plants, leaving the role of PSFs in plant invasions unclear (Bunn et al., 2015; Chiuffo et al., 2015; Schittko et al., 2016; Suding et al., 2013). Results from this study provide a clear example where a positive PSF was large enough to explain non‐native plant growth on the landscape.

While results were potentially consistent with previously reported patterns of PSF associated with succession and species origin (i.e., native or non‐native), perhaps a more parsimonious explanation for observed results was that PSF is positively correlated with plant abundance regardless of successional stage or native status (Klironomos, 2002). It is notable that the PSF effects observed in this study were derived almost exclusively from soil legacies created by the dominant native species and the dominant non‐native species. It is interesting to speculate as to why PSFs were observed only for the dominant species. It is possible that plants in this system only attained dominance if they were able to suppress dominant competitors. Species that fail to suppress the growth of other species through the soil were subdominant on the landscape.

Positive PSF was important to the communities in this study. Plants can create positive PSF in two ways: Plants can create soils that increase conspecific growth, or plants can create soils that decrease heterospecific growth (Bever, 1994; Bever et al., 1997). Both native and non‐native plant communities realized positive PSF by decreasing heterospecific growth. This was reflected in the fact that both native and non‐native communities grew similarly among most soils but poorly on one “other” soil treatment. Many mechanisms can explain this pattern. *Centaurea diffusa* may have decreased native growth by releasing allelochemicals (Callaway & Aschehoug, 2000; Quintana, El Kassis, Stermitz, & Vivanco, 2009), decreasing mycorrhizal abundance or effectiveness (Klironomos, 2002), or by increasing pathogen loading (Eppinga et al., 2006). Microbially mediated effects appeared more likely than allelopathy because a greenhouse experiment with *C. diffusa* and *P. spicata* found that soil effects on plant growth were observed in live but not sterile soil (Nolan, Kulmatiski, Beard, & Norton, 2015). Further, a paired experiment in the same field found clear differences between the bacterial, archaeal, and fungal communities in the soils created by *C. diffusa* and *P. spicata* (Kulmatiski et al., in press). Finally, it is also possible that native and non‐native plants created nutrient feedbacks. *Pseudoroegneria spicata* soils in a paired experiment in the same field demonstrated some of the slowest net N mineralization rates while *C. diffusa* soils demonstrated some of the fastest rates (Stark and Norton, 2015; Kulmatiski et al., in press). This could explain the slow growth of the early‐successional, non‐native community on *P. spicata* soils.

The native and non‐native communities both demonstrated positive PSFs in this experiment. In contrast, most PSFs reported in the literature are negative (Kulmatiski et al., 2008). Two factors that differed between this and many other studies were (i) relatively long‐term field measurements were used and (ii) communities rather than monocultures were used. Previous studies have found that field experiments tend to produce generally more positive PSFs than greenhouse experiments (Heinze et al., 2016; Kulmatiski et al., 2008) and that PSFs can accumulate over time (Hawkes et al., 2013). It is not known why field experiments would realize more positive PSF than greenhouse experiments, but this could reflect greater disease pressure in the greenhouse or greater facilitation in the field (Heinze et al., 2016). Plant communities may develop more positive PSFs than plant monocultures if competition or interspecies communication stimulates plant defenses or symbioses (Doornbos, van Loon, & Bakker, 2012; Harrison & Bardgett, 2010; Lee, Wood, & Lee, 2015; Shannon et al., 2012). Alternatively, plant communities may develop more positive PSFs than plant monocultures because dense monoculture growth may encourage the development of larger or more damaging pathogen populations (Burdon & Chilvers, 1982). Understanding of how PSFs function in communities and in field conditions remains a central and unresolved question (Casper & Castelli, 2007; Crawford & Knight, 2017; van Der Putten et al., 2013; Hendriks et al., 2013) but this research suggests that PSFs in communities in field conditions may be more positive than suggested by common greenhouse studies (Kulmatiski et al., 2008). It should be noted that PSFs were calculated somewhat differently in this study than most studies that rely on plant growth in monoculture. Here, soils cultivated by any member of a plant community (either native or non‐native) were considered “self” soils.

The PSF model predicted the general pattern of plant dominance on the landscape, but model predictions underestimated native growth and overestimated non‐native growth. One likely explanation for this is that the model did not include factors such as propagule pressure or biomass accumulation (Eppstein & Molofsky 2007; Hawkes et al., 2013; Kardol et al., 2013; Levine et al., 2006). In the model simulation reported here, propagule pressure was assumed to be equal for all species on all soils. This was performed to isolate PSF from propagule pressure effects on community composition, but under field conditions, propagule pressure is likely to be highly correlated with plant abundance. Correlating propagule pressure with plant abundance would improve model predictions of plant growth on the landscape (data not shown; Levine et al., 2006). Similarly, longer‐term simulations that allowed long‐lived plants to accumulate biomass can be expected to increase native abundance and decrease non‐native abundance on native soils over time.

Results suggest that manipulations of plant–soil interactions are likely to provide a powerful tool for managing plant communities (Nolan et al., 2015; de Voorde, Bezemer, Van Groenigen, Jeffery, & Mommer, 2014). Previous research at the study site has shown that soil treatments aimed at manipulating PSF (i.e., activated carbon addition) can increase native plant growth in non‐native soils (Nolan et al., 2015). Broadly, results suggest that an improved understanding and ability to manipulate plant–soil interactions can be expected to lead to the development of novel and powerful tools for managing plant invasions, diversity, productivity, and community composition (Compant, Duffy, Nowak, Clément, & Barka, 2005; Jeffery, Verheijen, van der Velde, & Bastos, 2011; Lehmann & Joseph, 2015; de Voorde et al., 2014).

## DATA ACCESSIBILITY

Data used in the manuscript are available as supporting information and by contacting the author.

## CONFLICT OF INTEREST

None declared.
